# Unsupervised User Similarity Mining in GSM Sensor Networks

**DOI:** 10.1155/2013/589610

**Published:** 2013-03-18

**Authors:** Shafqat Ali Shad, Enhong Chen

**Affiliations:** Department of Computer Science and Technology, University of Science and Technology of China, Huangshan Road, Hefei, Anhui 230027, China

## Abstract

Mobility data has attracted the researchers for the past few years because of its rich context and spatiotemporal nature, where this information can be used for potential applications like early warning system, route prediction, traffic management, advertisement, social networking, and community finding. All the mentioned applications are based on mobility profile building and user trend analysis, where mobility profile building is done through significant places extraction, user's actual movement prediction, and context awareness. However, significant places extraction and user's actual movement prediction for mobility profile building are a trivial task. In this paper, we present the user similarity mining-based methodology through user mobility profile building by using the semantic tagging information provided by user and basic GSM network architecture properties based on unsupervised clustering approach. As the mobility information is in low-level raw form, our proposed methodology successfully converts it to a high-level meaningful information by using the cell-Id location information rather than previously used location capturing methods like GPS, Infrared, and Wifi for profile mining and user similarity mining.

## 1. Introduction

Successful mobility profile building is the basis of a wide range of applications which includes viral advertisement systems [1, 2], potential warning systems [3], city-wide mapping and sensing [4], pollution detection and exposure [5], social networking, and community finding [6]. All of the mentioned applications are based on mobility profile building where a low-level raw mobility information is interpreted into a high-level meaningful information which can be utilized for useful purposes. As the mobility profile building is based on two potential parameters, that is, dwell time extraction and significant location finding, spatial data-based applications use the discrete location and continuous time information over mixed model.

As location extraction is a trivial task in mobility profile building, there are two broad classifications of location extraction methods: Active badge [7] and Active bat [8], where Active badge mainly represents the indoor technologies like Bluetooth, RFID, and Infrared, while Active bat represents the outdoor technologies like GPS, assisted faux GPS, and GSM. As Active badge is limited in terms of its usage and implementations, Active bat is popular for location extraction in mobility. In case of Active bat, GPS and assisted GPS are not so encouraging because of high power consumption and extra equipment installation in the network. So, the only available and suitable method is GSM [9], where cell global identity (CGI) can be used for readily extraction of location. Cell global identity is a four-set header, that is, mobile country code (MCC) varies with country of the operator, mobile network code (MNC) binds with every network operator, location area code (LAC) assigned and arranged by the network operator for cells arrangement, cell ID given to every user connected to the network. MCC, MNC, LAC, and cell ID as a whole identify the user over its unique location in the network anytime. 

CGI represents the approximate location of user through its four-set header which can be converted into latitude and longitude coordinates using public cell ID databases. This location information can be used for the determination for significant places for mobility profile building of the user. However, extraction of significant locations is a trivial task due to many reasons like missing values, cell oscillation, and exact coordinate mapping for location. Additionally, the semantic information about the locations visited by the user can also be used for the mobility extraction through its mapping with physical location coordinates.

As the low-level mobility data cannot be used for the high-level potential mobility applications, we introduced a complete framework in this paper to describe how this information can be used to develop a mobility profile using the unsupervised clustering approach. So, the paper presents the extraction of spatiotemporal mobility trends and mobility profile building approach using the cellphone low-level log data. The contributions of the paper are (1) missing values extraction and removal of outliers using public cell ID and semantic information, (2) cell oscillation resolution and extraction of significant locations, (3) extraction of significant locations using overlapped area over time span, and (4) semantic information usage for final mobility profile building and user's similarity finding.

## 2. Related Work

Over recent years, mobility data has become a rich source of human life trends, and a lot of work has been done in the area of spatial information extraction. This motive is the main basis for many applications like city-wide sensing [10–14], where the privately held sensors were used through a developed model, while personal sensors like mobile phones and cameras are used for traffic monitoring system [15] through capturing the location information, while social behavior is studied in their work [16–18] where information is exploited through identification of significant places where users are active and later similarity analysis between them. While route prediction and recommendation is studied by Hull et al. [19] through GPS installed sensors in a taxi using the technique of opportunistic message forwarding. On the other hand, their work [20–22] is a cell-based location awareness for user mobility analysis.

In their work, Zonoozi and Dassanayake [23] proposed time optimization technique over cell residence for human mobility analysis, while Markoulidakis et al. [24] proposed prediction model through cell handover residence based on Markov model by introducing Kalman filter for future visit prediction. Akyildiz et al. [25, 26] proposed a prediction model that is based on motion, speed, position, and history. Musolesi and Mascolo [27] categorized the mobility models into traces and synthetics, where they suggested that trace-based mobility models are more easy to implement as compared to synthetic based due to its public data gathering. González et al. [28] studied the spatiotemporal nature of user mobility based on pattern analysis through extraction of top K locations from mobility data of 100 K. Nurmi and Koolwaaij [29] proposed a clustering technique for the extraction of significant places using a graph-based transitional model over cell tower location data.

All of the above-mentioned work is related to the mobility analysis done over complete information about location without consideration of missing values and change in network structure fall under data preprocessing and focused on location extraction either all dependent on semantic awareness or otherwise ignoring the semantic information all together. In our work, we have tackled with data preprocessing, where outliers have been eliminated and data is made consolidated for analysis, and further cell oscillation issue is resolved for complete mobility profile building, then all this clustered information has been used for the mobility profile building through a proposed clustering technique which is a mixture of semantic information and GSM network property usage. Our work is mainly focused on successful mobility profile building based on a naive approach that is a mixture of both semantic information and raw location information, where prior to profile building outlier, extraction of location information from GSM cell global identity (CGI) and cell oscillation phenomenon are well dealt by experimenting them on MIT reality mining mobility dataset.

## 3. Methodology

**Figure 1:**
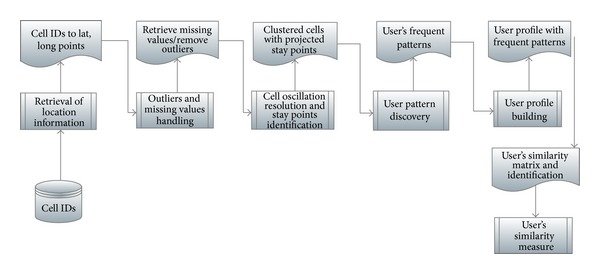
The proposed framework for user's similarity measure.

Figure 1 shows the overall process of proposed methodology.

### 3.1. Location Information Retrieval and Outlier Removal

**Figure 2:**
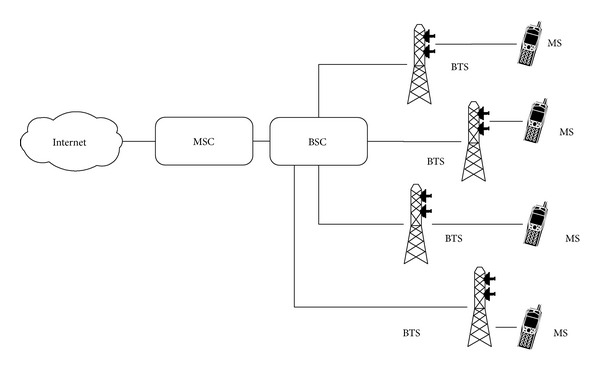
Architecture of functional GSM network.

The basic GSM structure is shown in Figure 2. As shown, the base transceiver station (BTS) is a basic unit being a representative of the location area where multiple cells fall in. This distribution is dependent on mobile operator and is hidden from users or public use. 

Mobile station (MS) moves in the network and gets its connection through base station controller (BSC). While BSC is connected to mobile services switching center (MSC), which connects different BSCs and MSCs over network. One important identity is the location area which all BSCs share connected to common MSC.

**Figure 3:**
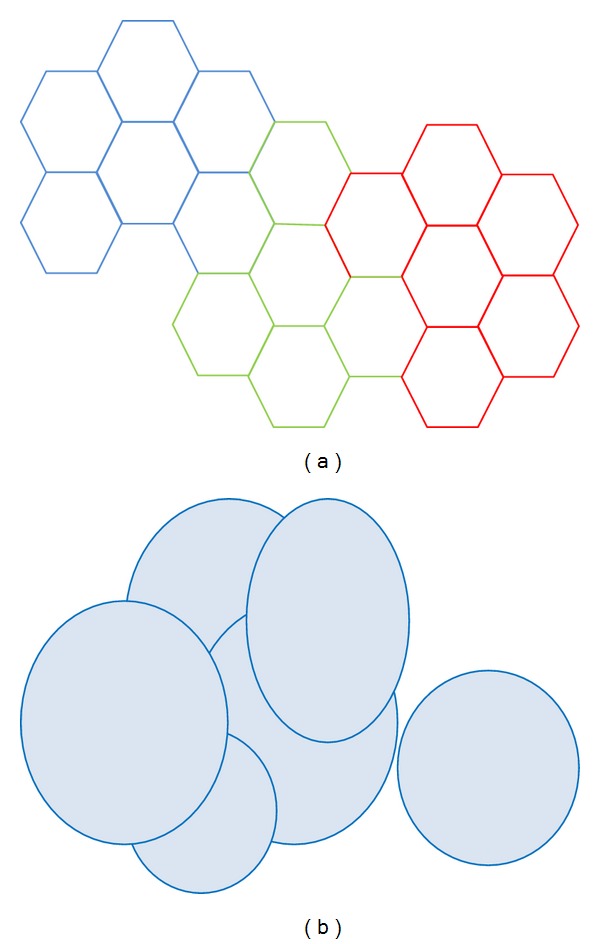
(a) Network topology: hexagonal view. (b) Cells overlapped view.

Each cell conceptually has polygon shape (Figure 3(a)), but actually it has overlapping bubble shape as shown in Figure 3(b).

Now there are two main concerns regarding the extraction of information from dataset; firstly as mentioned earlier the dataset is taken from MIT reality mining, which has partial information of cell global identity (CGI) about user location, that is, LAC and Cell ID, so it is apparently hard to determine whether this partial set of information is enough for location extraction; secondly the GSM network is changing over time, so LAC is reorganized or thrown away by the operator, so it is obvious that there will be missing values and outlier issues in dataset beside shift of GSM to 3G technologies nowadays which disable us from determining most of the location information using MIT dataset collected in 2008.

We proposed the methodology to deal with all these problems in our work [30], where we used the basic network information to solve the missing values issue and the outlier resolution along with precise clustering of cells for future location extraction and mobility profiling. We proposed and used the clustering methodology where LAC and cell ID provide all set of information for mobility profiling basic building using the open source Google location API reverse engineering. And for missing values, we utilized the semantic information provided by the user in the dataset for precise location extraction and mobility profile building. 

### 3.2. Cell Oscillation Resolution and Role of Semantic Information

As stated earlier in the problem statement, cell oscillation is a common phenomenon in GSM network, where a user can be assigned multiple cell IDs while static, which leads to a fake mobility during mobility profile building due to change in cell IDs over time. We presented a methodology in our work [31] for cell oscillation resolution using the semantic tagging information and introducing the time stay phenomenon, where overlapping cells represent the location of interest rather than mobility. In our mentioned work, we not only resolved the oscillation phenomenon successfully but also we clustered the cells on the basis of semantic information provided by user, for example, home, lab, airport, club, and so forth and overlapping time stay area, so that later during mobility profiling, this clustered information can be utilized for stay location identification. We used the overlapped location information for identification of significant places which can later be utilized for mobility profiling. 

### 3.3. Mobility Profile Building

Mobility profile building is one of the trivial tasks in any of the location base service (LBS), where the mobility profiling is done through extraction of significant places. The significant places can be defined as the places which are important for a user over geocoordinates, and most of the time the user stays on these locations. User usually semantically tags these locations or spends significant amount of time on these location or visits them frequently over the period of observation. So, it is clear that a significant place can be a place where user spends most of time (home, work, etc.) or user visits it frequently over a period of time (super market, club) or user spends time significantly without frequent visit (conference, seminar, travel). So, it makes the discovery of significant places valuable for LBS, where user behavior is the main source of stimulation. As described in previous sections, the cell ID is the only viable solution for user profile building, where the coverage is wide, low in energy consumption, no data plan is required, and available in all kinds of mobile phones, so user movement is available in data as set of different cell IDs are distributed over a network. By observing the precise transition over these cells and using the effective technique, this information can be used for mobility profile building. But construction of mobility profile building is a complex process, as it must deal with some of the following questions like when user moves over thousands of cell IDs during period of time many of them cannot be available to extract their location information through cell ID databases, for example, Google, Open Cell ID, so these cells will lead to misinterpreted profiling; there are dark places where user lost the connection or user switched off the cell, which seems to be significant places due to time spent by the user, and there is a lot of cell oscillations during user movement, which seems to be mobility even when user is static. We have divided it into three parts (1) clustering of the cells for path finding and their fingerprinting, (2) grouping of similar patterns for projection of trends, (3) profile building, and (4) similarity measure between different users through sharing property as adopted from [32]. The whole process of mobility profiling can be elaborated as follows.

Let *T* = {*t*_1_, *t*_2_,…, *t*_*n*_} is set of towers/locations visited by the user during the mobility, we are interested in identification of pattern group PG = {pg_1_, pg_2_,…, pg_*n*_}, which satisfies the TH_groupcount_ which is group threshold, where PG is extracted from frequent mobility user history (FUH) defined over transition threshold TH_transition_, location area threshold TH_loctaion  area_, and semantic tag information SEM. FUH is retrieved through the visiting history VH retrieved through oscillation removal method [31] and time-stamping methods.

**Algorithm 1**

Frequent pattern discovery from user's mobility history.

1. Select complete list of user mobility in terms of cell towers visited *T*.
2. Apply cell oscillation technique on it and retrieved clustered cells *C*.
3. Apply proper time stamping on the clustered cells retrieved after oscillation removal as visit history of user VH with complete spatiotemporal information.
4. Identify the frequent user mobility patterns FUH defined over TH_loctain  area_ and TH_transition_. Group the identified patterns G on the basis of their spatiotemporal nature and semantic tags information using prefix-span algorithm.
5. Repeat step (i) to (ii) until only supported group of patterns PG is identified
  - if size of group *g* satisfies the group support threshold TH_Gorupcount_,
  - assign the group to supported group pattern set.

Let the set of users *U* = {*u*_1_, *u*_2_,…, *u*_*n*_} with the complete information of pattern groups PGs, we are interested to build an M[*i*][*j*] on the basis of each set of patterns *p*_*i*_ and *q*_*j*_, where two patterns belong to two potentially similar users. The similarity between two patters is calculated over LnCSS (longest common subsequence) and CoL (colocation probability measure).

**Algorithm 2**

User's similarity measurement.

1. Select the users.
2. Select the pattern group of two users.
3. Repeat step (i) to (ii) for each *p* and *q* belongs to two different users
  - calculate the LnCSS and CoL property of two given patterns,
  - if two patterns satisfy the minimum support of similarity than compute M(*p*, *q*) as 1 otherwise compute it as 0.
4. Calculate the user's similarity based on similarity matrix.

#### 3.3.1. Clustering of Subsequences in Mobility History Data

As mentioned earlier, we retrieved the clustered cells by implementing the cell oscillation algorithm where it is guaranteed that the data has no oscillation problem. We adopted naïve clustering approach, which clusters the cells on the basis of circular subsequences. The baseline algorithm works on the fact that common cell IDs can be merged together to make a circular subsequence which can represent user behavior [31]. We implemented two proposed algorithms successfully [31] for the resolution of cell oscillation and retrieval of common clustered cells to represent the significant place for the user using the overlapped area; however, details of the algorithm are given in our previous work. It is also of due importance that as mentioned earlier the proposed algorithm also resolved the problem of missing values which can occur due to network change or nonavailability of cell-ID information in open cell-ID databases. For example, the mobility sequence *C*_1_, *C*_2_, *C*_3_, *C*_4_, *C*_5_ has *C*_3_ and *C*_4_ no retrievable through cell-ID database which obliviously will lead to a mislead mobility of the user; our algorithm will replace these two cells using the majority voting mechanism with the most likely cell in the cluster, so that mobility remains traceable with the most likely authentication. The retrieved clusters carry the information about significant places as a semantic tag like home, lab, club, and so forth, as the derived algorithm can infer the significant place based on time spent over an overlapped area between different cells; we tagged such location with a convention of “*stay point*”. After the retrieval of the clustered cells, we implemented the fingerprinting on them to represent the percentage of time spent by the user on particular cells and as a whole on one cluster, that is, stay points. Each cluster is being represented as a sequence of cell IDs, that is,

(1) Cluster1[C1,C2,C15],Cluster2[C3,C5,C7,…,C19]⋮Clustern[C7,C18,…,C24].

Each cluster represents the stay point which may or may not have user defined semantic tag associated with it.

#### 3.3.2. Time Stamping of Clustered Cells

We bound the time percentage with each of the cells in the cluster along with total time spends on that particular cluster with time stamp. The time stamping is the most important part of fingerprinting, where user behavior can be determined easily over time/date slicing. The structure of fingerprint is as

(2) [{{C1,%T1},{C2,%T2},…,{Cn,%Tn}}, Total.time,Time  stamp,Semantic  tag],

where each cluster represents the stay point of the user along with percentage time on it as %*T*, date, and semantic tag information if available. After this process, we have a set of all the stay points or significant places user had visited over time, so we converted the given history of user distributed over cell IDs to stay points in form of clusters as follows:

(3) $$\begin{array}{c} \text{VH}=\left\{{\text{Cluster}}_{1},{\text{Cluster}}_{2},\ldots ,{\text{Cluster}}_{n}\right\}, \end{array}$$

where VH represents the complete mobility history of the user in terms of stay points in form of spatiotemporal clusters, that is, Cluster_1_, Cluster_2_,…, Cluster_*n*_. This information can be used to identify the Trajectory patterns against a particular user to build up the mobility profile by grouping them distributed over time. After the extraction of patterns, we need to group the patterns and discard infrequent pattern through usage of minimum support count otherwise the resultant pattern will be in millions for such a huge amount of data, which will be a burden in the memory. So, we are interested in only frequent pattern through usage of spatiotemporal information and support count. So, we can define group G as subset of VH such that it satisfies the following two rules:

- *P*_1_, *P*_2_,…, *P*_*n*_ ∈ *g* if *g* · Distance ≤ TH_location  area_ and *g* · Time ≤ *g* · TH_stay  time_
- |*g*| ≥ TH_group  support_.

#### 3.3.3. Extraction of Mobility Patterns from Clustered Stay Points

After the retrieval of all the stay points in the given spatiotemporal history of mobile user, we can transform it into trajectory pattern which the user follows over time. We can define a pattern *P* as a trip over two or more consecutive stay points SPs in ordered set of VH or trip between stay point SP and first/last point of user mobility history VH. We can define the pattern *P* as

(4) P={SPm,…,SPn}, where  0<m≤n,Pi(VHi{…,SPm}∩VHi+1{SPn,…}) OrP={SP1,…,SPm}, where  0<m≤o,Pi(VHi{SPm,…  }) OrP={SPn,…,SPo}, where  0<n≤o,Pi(VHi{…,SPn}).

Further for the extraction of the true patterns from user mobility, we introduced the transition time threshold to ensures the continuity of the trip, where this transition threshold ensure the smooth transition of user from one stay point to another and differentiates one visiting pattern from another. For the extraction of mobility pattern from the semantically arranged stay points, we used prefix-span algorithm [33] on user mobility history. The result of algorithm gave semantic pattern of the user over time, for example, 〈{Home}, {Lab, Google}, {Grand  parents}〉 along with all of its subsequences. As the result of the algorithm gave redundant patterns, we adopted maximal trajectory pattern [34] technique for the representation of user mobility pattern. So, the resultant extracted patterns are true representatives of frequent mobility of user (FUM) called over time as

(5) FUM=  {Ptn1:  〈{Home},{Stay  point},{Office,Google},          {Stay  point  }〉,     Ptn2:  〈{Home},{Super  market,Topo  hub},         {Grand  parents,Club}〉,…,     Ptnn:  〈{Office,Bank},{Stay  Point,Park}〉}.

#### 3.3.4. Projection of Similarity between Different Users Through Pattern Matching

After the successful extraction of user pattern, we determine if two patterns are similar through the longest common subsequence (LnCSS) measure. For example, if there is pattern *P* = 〈{Home}, {Stay  point}, {Office, Google}, {Stay  point}〉 and *Q* = 〈{Home}, {Office, Bank}, {Stay  point}〉, their LnCSS will be = 〈{Home}, {Office}, {Stay  point}〉, which we can define as

(6) Ratio (
LnCSS
(p,q),p) =∑i=1|p|∑j=1|LnCSS(p,q)|M(pi,LnCSSj)|p|M (pi,LnCSSj)=pi∈LnCSSj|p|  if  LnCSSj  is  matching  to  pi  otherwise=0.

But the longest common subsequence (LnCSS) measurement is not only based on merely semantic tag based as same place is tagged with different names against different users, for example, Media Lab is tagged as Lab, Media Lab, Work lab, and so forth, so we introduced the time threshold for the transition, that is, TH_trans⁡_ between two stay points (as every semantically tagged location consists of cell towers); further we introduced the spatial property threshold using location TH_location  area_ area, so it together joins the spatiotemporal property of user mobility. 

So, we can define the similarity between two users; given two frequent user mobility (FUH) FUH_1_ = {Pattern_1.1_, Pattern_1.2_,…, Pattern_1.*n*_} and FUH_2_ = {Pattern_2.1_, Pattern_2.2_,…, Pattern_2.*n*_} and defined time threshold TH_time  cover_ and location area threshold TH_location  area_, we can define similarity between users as

(7) Similarity (FUH1,FUH2,THtime  cover,THlocation  area) =Location  area  (Ptn1.1,Ptn2.i)  +Distance  (Ptn1.n,Ptn2.j≤THlocation  area),CoL (Ptn1.1,Ptn2.i)≤1, where    i,j  belongs  to  N ∣ 0<i≤j≤m,

where CoL is colocation property of that find is user *x* and *y* visit location *l* at the same time to exploit their spatiotemporal property beside semantic tags; as mentioned, semantic tags cannot assure the true accuracy of similarity measure due to different conventions used by different users for the same place. Colocation rate idea is adopted from their work [35], where the most likely location of the user *x* is defined as

(8) $$\begin{array}{c} L\left(x\right)=\text{ar}{\text{g}}_{l\in \text{Loc}}P\left(x,l\right), \end{array}$$

where *L* is the most likely location of user *x*, and Loc represents the cell towers or locations set user traversed over time during mobility, while *P* is the probability of the user *x* to visit location *l* and can be defined as

(9) $$\begin{array}{c} P\left(x,l\right)=\sum_{i=1}^{n(x)}\frac{\delta \left(l,L_{i}\left(x\right)\right)}{n\left(x\right)}, \end{array}$$

where *δ*(*x*, *y*) = 1 if *x* = *y* otherwise it is 0. Further distance between user *x* and *y* can be defined as *d*(*x*, *y*) = dist⁡(*L*(*x*), *L*(*y*)) to represent physical distance between their frequent locations. So, on the basis of it colocation rate can be defined as follows:

(10) CoL= ∑i=1n(x)∑j=1n(y)Θ(ΔT−|Ti(x)−Tj(y)|)δ(Li(x)−Lj(y))∑i=1n(x)∑j=1n(y)Θ(ΔT−|Ti(x)−Tj(y)|).

As (10) shows that colocation rate counts both time and location simultaneously for two users, that is, *x* and *y*. So, it binds both spatiotemporal trends together normalized over the number of times the users visited the location, where Θ is Heaviside function and Δ*T* is equal to *T*_stay time_.

**Figure 4:**
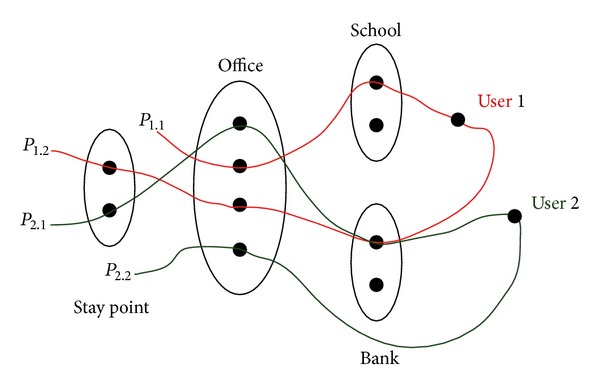
User mobility pattern.

On the basis of the above similarity measuring value, we can calculate the similarity between the given user pairs through comparing their patterns over three basic units: semantic tag, spatial value, and temporal value. For this we construct a similarity matrix which gives a clear picture of similarity measure against every frequent pattern pair of two different users. After calculating the similarity between patterns of two users, these values are used to calculate the overall similarity measure between two users to infer if they are related to each other or not. For example, we have two user's   FUH_1_ = {*P*_1.1_, *P*_1.2_} and FUH_2_ = {*P*_2.1_, *P*_2.2_} to find their similarity with a given pair of patterns, where *P*_1.1_ = {〈Home〉〈Bank, Office〉〈Stay  point〉} and *P*_1.2_ = {〈Home〉, 〈School〉, 〈Office〉}, while *P*_2.1_ = {〈Home〉〈Bank, Office〉〈Stay  point〉} and *P*_2.2_ = {〈Home〉, 〈Office〉}.Figure 4 shows the spatial incidence of these patterns. We can construct a similarity matrix through it as shown in Table 1.

So, the similarity measure can be concluded as = Sum of all extracted similarity weights/Total number of patterns, where the high value represents the most similarity among two users, while the lower value represents the dissimilarity between the two users. 

So, on the basis of the above similarity measure, we can define the profile sharing measurement of the given users as

(11) Sharing  value (FUH1,FUH2,THlocation  area,THtransition)  =|{p∈FUH1 ∣ ∃q∈FUH2·similarity(p,q,CoL)}|  |FUH1|.

## 4. Dataset

As mentioned earlier, the selected dataset is taken from mining project group of MIT Media labs [36]. This dataset is collected from 100 people who are students for a period of 9 month with total activity span of 350 K hours. The collected data is logged on Symbian mobile, that is, Nokia 6600 which has no GPS in it, so all of the information related to user location is identified by cell ID only. While in this dataset, cell global identity header has partial information, where only LAC and cell ID are available for location tracking of the user. However, users have provided semantic tagging information to the most important locations over mobility history in data logs. But overall, this semantic tag information varies a lot in terms of annotations and usage from user to user. And among these users only, 94 gave their full information regarding similarity measure through online survey for social interactions. From these 94 users, 7 of the users do not have cell logs and 10 have no cell annotation logs. So, for our analysis there are only 77 users available for analysis and evaluation.

## 5. Experiments and Results

As mentioned, we have chosen the reality mining dataset for our experimental purposes. The results are as follows.

### 5.1. Retrieval of Location Information and Removal of Outliers Using Raw Cell ID

As the data is quite old in its nature and there are frequent changes in mobile network, so presences of outliers and missing values are obvious in the dataset, so we applied our clustering approach [30] on the raw data to remove spatial outliers from the data and extract their location information through Google APIs [30]. The result of applied technique is shown in Table 2.

**Figure 5:**
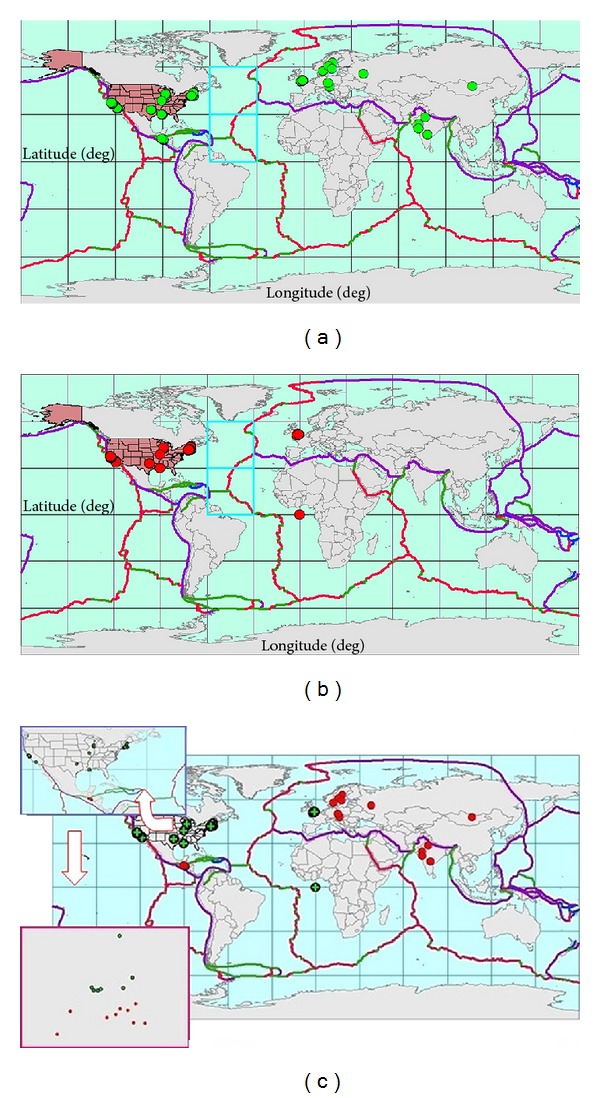


Figure 5(a) shows the cells retrieved from the Google API, and Figure 5(b) shows the consolidated data without outliers, while Figure 5(c) shows the complete effect of spatial clustering over the data.

### 5.2. Observation of Semantic Tags in Dataset

After the extraction of outlier free data points, we applied our spatial clustering techniques [30, 31] on the clean data to cluster them in terms of stay points which may or may not have semantic tags in them. As Table 2 shows that each semantic location observed can carry multiple cell IDs, so these cells can be clustered together to define a common location. As most of the time stays at known places are usually tagged, so these semantic locations are of more importance than untagged stay points [31], as shown in Table 3.

### 5.3. Cell Oscillation Resolution and Discovery of Stay Points

As defined previously cell, oscillation is a phenomenon obvious in GSM dataset, where user is assigned multiple cell IDs while being stationary for load balancing. Table 2 shows that semantic location can be identified with multiple cell IDs which make it clear that beside the semantic tag information, we are bound to use spatiotemporal analysis with location for pattern building and finding similar users, as this semantic tag is limited in the data. We have applied our spatial clustering technique [31] on the dataset for the removal of cell oscillation, and using the overlapping area analysis, we identified stay points which are not semantically tagged by the user otherwise. And as per our previous assumption, GSM cells are distributed in bubble form that they overlap with each other; this assumption is evident from resultant Table 4. 

**Figure 6:**
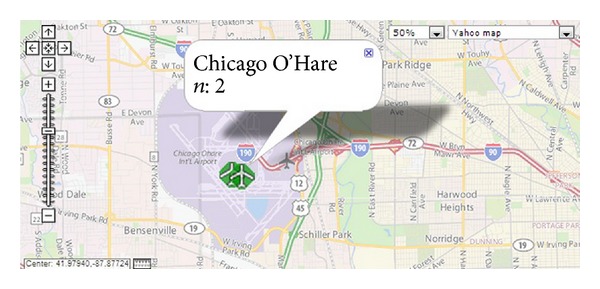
Semantic location plotted over geographical map.

As result we retrieved all the locations as a clustered cells which are representative of user mobility history rather the raw cells. In Figure 6 of the tagged locations are plotted over geographical map which shows the vicinity of tagged places, further Figure 6 shows our assumption is correct as Chicago O'Hare airport is tagged twice with overlapped cells by the user which shows overlapping of cells and oscillation over same place.

### 5.4. Discovery of Mobility Patterns

**Figure 7:**
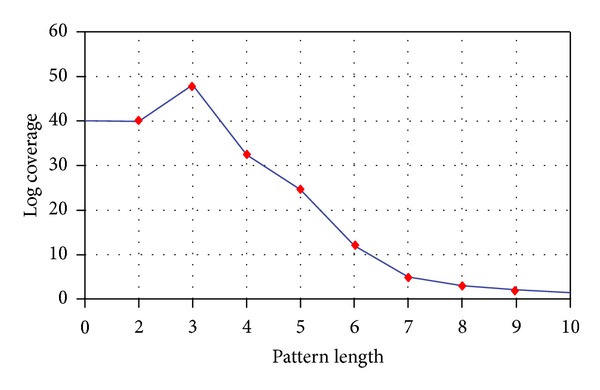
Mobility pattern length over log.

After the extraction of mobility history in form of clustered cells where each cluster represents the stay point with or without user defined semantic tag, we applied time-stamping methodology on it defined in Section 3.3.2. On this time-stamped clustered history, we applied the mobility pattern extraction technique proposed in Section 3.3.3. We used the TH_stay  time_ of 20 minutes along with TH_transition_ of 10 minutes which are adopted form our work [31]. After extraction of these mobility patterns, we implemented the maximal trajectory pattern technique proposed in Section 3.3.3 for the representation of the most frequent mobility pattern. We plotted graph in Figure 7 representing the log time coverage of the user mobility with respect to the length of pattern to analyze the most frequent pattern length. 

The figure clearly shows that most of the patterns which cover the log of user mobility are of length 3, after which the coverage is declined tremendously. This result gives information about the transition phase, length set for LnCSS, and trend of user over time, which we will be using later during similarity measure.

**Figure 8:**
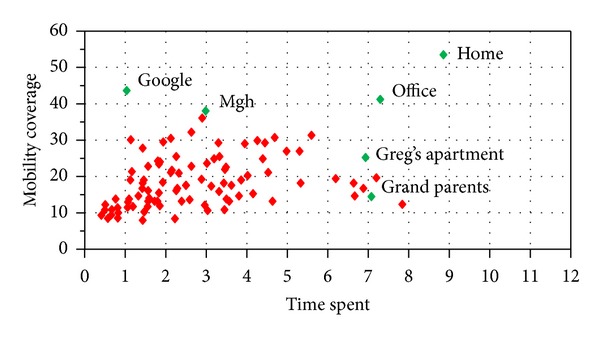
Locations visit over log.

As the explored patterns carry all the basic information like location, semantic tag, and time stamping, we can plot the user trend quite easily. As shown in Figure 8, we plotted the user location visit history against log history period, and we have selected top locations only due to of space limitation. We selected only two-month user history for this plotting. The figure clearly shows that user spent most of his time at known places and rarely explored new places, and this also shows one important fact about mobility data that user spent most of his time on the location which he tagged semantically, that is, home, MGH, office, and so forth. This satisfies our assumption in Section 3 about trend of the use mobility. This trend also shows some facts like that the user visits some locations like home everyday regardless of weekdays and weekend and some locations like office every weekday only, whereas some locations like Greg's home and grandparents user usually visit once in a week and on weekends.

**Figure 9:**
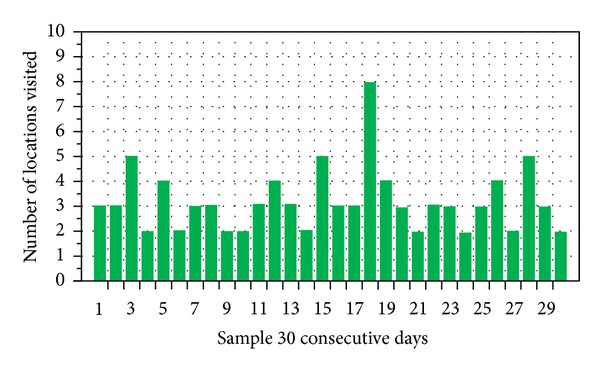
User's locations visit over days.

We plotted user mobility ratio in term of exploration with time for further analysis in Figure 9, which shows that user visit average is of 2–5 places daily, while in exception cases user visited more than 7 locations at a single day. We plotted the location visiting frequency over a data of 30 consecutive days.

### 5.5. Similarity Measure between Users

For the calculation of similarity measure, we constructed the similarity matrix on the basis of two main parameters, that is, semantic pattern and spatiotemporal similarity. For this we use LnCSS and set the TH_location  area_ to 10 being a Euclidean distance between the two points of location that belong to two different users to ensure if they fall in same area or not; further we set the TH_timecover_ as 20 minutes as described in Section 3.3.4. 

After formulating the similarity matrix, we plotted similarity measure between user *x* and other users along with other user similarity measurement models, that is, spatial cosine similarity (SCS) and extra-role colocation rate (ERCR) [37] for evaluation. Spatial cosine similarity can be defined as similarity of visitation frequencies of user *x* and *y* assigned by cosine of angle between the two vectors with respect to number of visit at each location. And extra-role colocation is based on probability of two users *x* and *y* to colocate in the same hour at night or on weekends. This relationship serves as a great indicator to determine the friendship between two users. 

We plotted the similarity measure between users using our proposed methodology named HA (Hybrid approach), SCS, and ERCR using the two metrics, that is, mean average precision (MAP) and normalized discounted cumulative gain (NDCG). 

**Figure 10:**
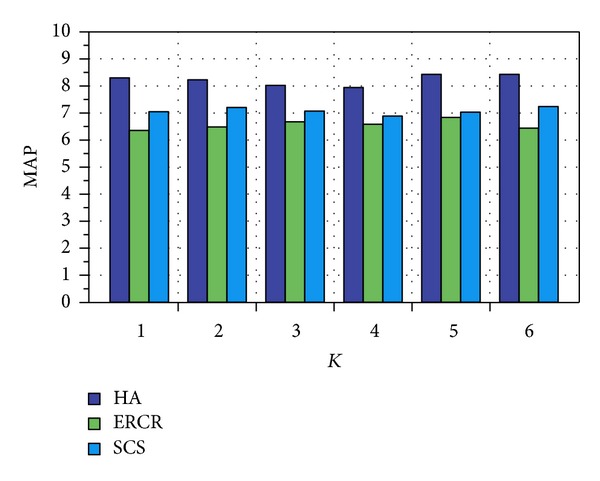
MAP with respect to proposed methodology performance.

In Figure 10, we plotted the user similarity based on MAP matrices where MAP can be defined as

(12) $$\begin{array}{c} \text{MAP}=\frac{1}{N}\sum_{i=1}^{N}\frac{\sum_{T=1}^{K}\left(P_{i}\left(r\right)\times {\mathrm{rel}}_{i}\left(r\right)\right)}{\left|R_{i}\right|}, \end{array}$$

where *N* is number of test users and |*R*_*i*_| indicates number of similar users for test user *x*, *r* donates cut-off rank and *P*_*i*_(*r*) represents precision of *U*_*i*_ over binary function *rel*⁡_*i*_(*r*).

**Figure 11:**
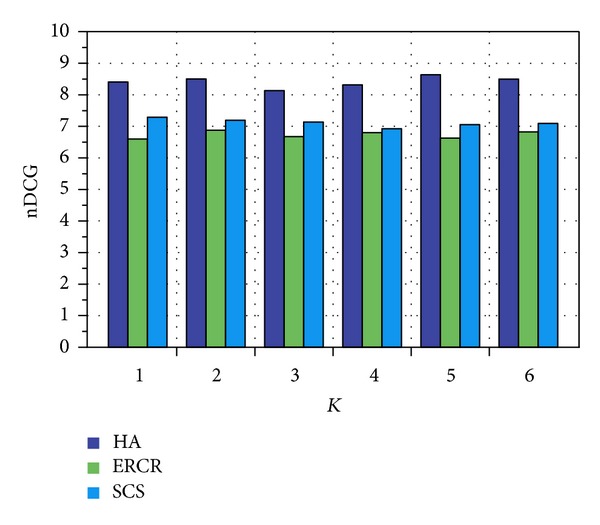
nDCG with respect to proposed methodology performance.

We plotted the similarity measure between users using the NDCG matrices as defined by [38] in Figure 11. 

Figures 10 and 11 clearly show that our proposed methodology outperforms with respect to spatial cosine similarity (SCS) and extra-role colocation rate (ERCR) over the indicated matrices. The experiments show that our proposed methodology outperforms on real dataset of MIT as compared to other mentioned methodologies.

## 6. Conclusion and Future Work

In this paper, we presented the two phases methodology of building mobility profile and finding user similarity, which is unsupervised approach based on the semantic tag information along with the spatiotemporal trends. As our methodology uses both semantic and spatiotemporal trends, this makes it outperform over the mere use of tag, spatial- or spatiotemporal-based methodologies defined earlier. As discovering the similar user can play a vital role in many potential applications related to location-based services (LBS), so our approach is quite efficient where semantic tag along with spatiotemporal trends can serve as a precise approach towards similarity measurement between different users. Further, this methodology is complete framework which resolves all mobility profiling issues, that is, outlier detection, missing values retrieval, cell oscillation, user trajectory profiling, and similarity measure.

Future studies can be carried out on the behavior of users depending on transient reactions like civil work on road, and a special event together with their trajectories to determine the exact behavior of user for its application in real scenarios for location-based services.

## Figures and Tables

**Table 1:** Similarity matrix.

	*P*_1.1_	*P*_1.2_	*P*_2.1_	*P*_2.2_
*P*_1.1_	—	—	1	0
*P*_1.2_	—	—	0	1
*P*_2.1_	1	0	—	—
*P*_2.2_	0	1	—	—

**Table 2:** Locations retrieved with respect to user mobility.

	# of cells	%
Total number of unique cells against subject X	1744	100%
# of cell's location retrieved through Google API	680	39%
# of cell's location retrieved through semantic tagged algorithm	88	3%
Total # of cell's location retrieved	768	42%

**Table 3:** Semantic location with # of representative cells.

Semantic location	Number of cells incidence
Mgh	5
Office	4
Airport	4
Home	4
Greg's apt	4
Grand parents	3
Google	3
Redhat	3
Chicago O'Hare	2
Topo hub	2

**Table 4:** Overlapping of locations.

Cells (LAC. Cell ID)	# of locations represented
*C*_30_	7
*C*_40_	6
*C*_23_	6
*C*_14_	5
*C*_56_	4
*C*_44_	4
*C*_25_	3
*C*_47_	2
*C*_27_	2
*C*_39_	2
